# Use of Disease-Modifying Therapies in Pediatric Relapsing-Remitting Multiple Sclerosis in the United Kingdom

**DOI:** 10.1212/NXI.0000000000001008

**Published:** 2021-05-21

**Authors:** Omar A. Abdel-mannan, Celeste Manchoon, Thomas Rossor, Justine-Clair Southin, Carmen Tur, Wallace Brownlee, Susan Byrne, Manali Chitre, Alasdair Coles, Rob Forsyth, Rachel Kneen, Kshitij Mankad, Dipak Ram, Siobhan West, Sukhvir Wright, Evangeline Wassmer, Ming Lim, Olga Ciccarelli, Cheryl Hemingway, Yael Hacohen

**Affiliations:** From the Queen Square MS Centre (O.A.A., W.B., O.C., C.H., Y.H.), UCL Queen Square Institute of Neurology, Faculty of Brain Sciences, University College London; Department of Neurology (O.A.A., O.C., C.H., Y.H.), Great Ormond Street Hospital for Children, London; Children's Neurosciences (C.M.), Evelina London Children's Hospital, Guy's and St Thomas' NHS Foundation; Department of Paediatric Neurology (T.R., M.C.), Addenbrooke's Hospital, Cambridge; Department of Neurology (J.-C.S., R.K.), Alder Hey Children's NHS Foundation Trust, Liverpool, United Kingdom; Queen Square Institute of Neurology (C.T.), Faculty of Brain Sciences, University College London; Multiple Sclerosis Centre of Catalonia (Cemcat) (C.T.), Vall d’Hebron Institute of Research, Vall d’Hebron Barcelona Hospital Campus, Spain; Children's Neurosciences (S.B.), Evelina London Children's Hospital, Guy's and St Thomas' NHS Foundation Trust, King's Health Partners Academic Health Science Centre, London; Department of Clinical Neurosciences (A.C.), Addenbrooke's Hospital, Cambridge; Translational and Clinical Research Institute (R.F.), Newcastle University; Department of Neuroradiology (K.M.), Great Ormond Street Hospital for Children, London; Department of Neurology (D.R., S. West), Royal Manchester Children's Hospital, Manchester; Department of Neurology (S. Wright, E.W.), Birmingham Children's Hospital, Birmingham; Aston Neuroscience Institute (S. Wright, E.W.), College of Health and Life Sciences, Aston University, Birmingham, United Kingdom; Evelina London Children's Hospital (M.L.), Guy's and St Thomas' NHS Foundation Trust, King's Health Partners Academic Health Science Centre, London, United Kingdom; and NIHR University College London Hospitals Biomedical Research Centre (O.C.).

## Abstract

**Objectives:**

To compare the real-world effectiveness of newer disease-modifying therapies (DMTs) vs injectables in children with relapsing-remitting multiple sclerosis (RRMS).

**Methods:**

In this retrospective, multicenter study, from the UK Childhood Inflammatory Demyelination Network, we identified children with RRMS receiving DMTs from January 2012 to December 2018. Clinical and paraclinical data were retrieved from the medical records. Annualized relapse rates (ARRs) before and on treatment, time to relapse, time to new MRI lesions, and change in Expanded Disability Status Scale (EDSS) score were calculated.

**Results:**

Of 103 children treated with DMTs, followed up for 3.8 years, relapses on treatment were recorded in 53/89 (59.5%) on injectables vs 8/54 (15%) on newer DMTs. The ARR was reduced from 1.9 to 1.1 on injectables (*p* < 0.001) vs 1.6 to 0.3 on newer DMTs (*p* = 0.002). New MRI lesions occurred in 77/89 (86.5%) of patients on injectables vs 26/54 (47%) on newer DMTs (*p* = 0.0001). Children on newer DMTs showed longer time to relapse, time to switch treatment, and time to new radiologic activity than patients on injectables (log-rank *p* < 0.01). After adjustment for potential confounders, multivariable analysis showed that injectables were associated with 12-fold increased risk of clinical relapse (adjusted hazard ratio [HR] = 12.12, 95% CI = 1.64–89.87, *p* = 0.015) and a 2-fold increased risk of new radiologic activity (adjusted HR = 2.78, 95% CI = 1.08–7.13, *p* = 0.034) compared with newer DMTs. At 2 years from treatment initiation, 38/103 (37%) patients had MRI activity in the absence of clinical relapses. The EDSS score did not change during the follow-up, and only 2 patients had cognitive impairment.

**Conclusion:**

Newer DMTs were associated with a lower risk of clinical and radiologic relapses in patients compared with injectables. Our study adds weight to the argument for an imminent shift in practice toward the use of newer, more efficacious DMTs in the first instance.

**Classification of Evidence:**

This study provides Class IV evidence that newer DMTs (oral or infusions) are superior to injectables (interferon beta/glatiramer acetate) in reducing both clinical relapses and radiologic activity in children with RRMS.

Pediatric multiple sclerosis (MS) has an incidence ranging between 0.13 and 0.66 per 100,000 children per year.^1^ In comparison to MS in adults, pediatric MS is associated with a higher relapse rate^2^ and rapid MRI lesion accrual early in the disease course,^3^ with worse cognitive outcomes^4^ and physical disability in the long term. Furthermore, patients show progressive brain atrophy, especially in the gray matter.^5^ Despite a highly active disease, particularly in the initial years, patients with pediatric-onset MS demonstrate a slower rate of accrual of disability compared with adult-onset patients.^6^

The approach to treatment of relapsing-remitting multiple sclerosis (RRMS) is rapidly evolving, with 15 disease-modifying therapies (DMTs) currently licensed for adults, that target the immune system peripherally and reduce MS relapse risk.^7^ These range from injectables (interferon-β and glatiramer acetate), with favorable side effect profiles but moderate clinical efficacy, to newer oral or infusion DMTs, associated with greater treatment efficacy, but a higher risk profile.^8^ Current treatment strategies in pediatric MS are largely center specific and reliant on adult protocols; there is a clear need to balance the risk of undertreating children causing poor disease control and accrual of motor, visual, and cognitive disabilities or overtreating them, particularly with sequential immunotherapy, exposing the child to unnecessary toxic effects, which may include reduced fertility, malignancy risks, and premature immune senescence.^9^ Nevertheless, an improvement in prognosis with a globally reduced annualized relapse rate in children with MS is observed compared with the pretreatment era,^10^ indicating a possible long-term effect of therapies. In a multicenter observational study of 741 pediatric patients with MS from the US Network of Pediatric MS Centers, those on newer DMTs had lower relapse rates (rate difference = 0.27, *p* = 0.004) and lower rates of new/enlarging T2 (hazard ratio [HR] = 0.51, *p* < 0.001) and gadolinium-enhancing lesions (HR = 0.38, *p* < 0.001) than those on injectables.^11^ The results of the first pediatric MS randomized controlled trial of fingolimod (newer oral DMT) vs interferon-β (older injectable DMT) demonstrated the superiority of fingolimod over interferon-β, with a lower relapse rate, less accumulation of lesions on MRI, and a lower annualized rate of brain atrophy over a 2-year period.^12,13^

There are very few studies to guide optimal initial DMT choice for pediatric MS. We therefore aimed to evaluate real-world effectiveness of treatment with newer compared with injectable DMTs in children with RRMS. In addition, we aimed to compare the different clinical (annualized relapse rate [AAR]), imaging (≥2 new T2 hyperintense and/or ≥1 gadolinium-enhancing lesions), and disability parameters (Expanded Disability Status Scale [EDSS] score) before and on treatment.

## Methods

This project was a multi-institutional, retrospective study run within the UK Childhood Inflammatory Demyelination Network, involving 7 pediatric neuroscience centers commissioned to manage pediatric-onset MS: Great Ormond Street Hospital (London), Evelina London Children's Hospital (London), Birmingham Children's Hospital, Addenbrooke's Hospital (Cambridge), Alder Hey Children's Hospital (Liverpool), Royal Manchester Children's Hospital, and Great North Children's Hospital (Newcastle). Centers were asked to identify patients aged <18 years with RRMS receiving DMTs in the period of January 2012 to December 2018, using Blueteq records of DMT prescriptions (drug management system used by NHS England for high-cost drugs including DMTs).^14^ Patients who entered a clinical trial with a DMT were excluded. Clinical data including demographics, clinical findings and laboratory results, first and subsequent relapse characteristics, and treatment information were retrospectively reviewed from electronic medical records of patients and entered into a standardized database.

All patients had undergone brain imaging according to local MRI protocols every 6 months with a new baseline MRI once commenced on new treatment. Spinal cord imaging was only performed when clinically indicated and was not performed routinely.

The patient's age at DMT start, year the DMT was prescribed, duration of use, side effects, and reasons for discontinuation or switching therapies were included. The NCI Common Terminology Criteria for Adverse Events (v.5) were used for reporting of adverse events using a grading (severity) scale.^15^ Markers of disease severity included first relapse characteristics (polysymptomatic, transverse myelitis, and optic neuritis), number of relapses and presence of new T2 hyperintense or gadolinium-enhancing lesions on brain MRI before treatment and on treatment, and EDSS scores at baseline and last follow-up.

DMTs classified as newer included dimethyl fumarate, fingolimod, teriflunomide, natalizumab, ocrelizumab, and alemtuzumab. DMTs were also classified by mode of administration, including injectables (glatiramer acetate and interferon-β), oral (dimethyl fumarate, fingolimod, and teriflunomide), or infusion (natalizumab, ocrelizumab, and alemtuzumab). The primary comparison was between patients on injectables and those who were started on or escalated to newer DMTs (oral and infusions).

The following outcome measures were calculated: (1) ARRs before and on treatment, (2) time to clinical relapse from treatment initiation (relapses are defined as new/worsening neurologic symptoms lasting at least 24 hours in the absence of fever or infection, as determined by the treating neurologist), (3) time to switching DMT from treatment initiation, (4) time to development of ≥2 new T2 hyperintense and/or ≥1 gadolinium-enhancing lesions on brain MRI from treatment imitation, and (5) change in EDSS score from baseline to last follow-up on treatment.

ARRs were calculated as the number of relapses per year before treatment (excluding index event) and during treatment only in patients with at least 6 months of follow-up after initiation of treatment. An attack was defined as “definitely new neurologic symptom” or “clear acute worsening of previous neurologic deficits” with objective clinical signs, lasting for at least 24 hours and attributed to an inflammatory CNS event, confirmed by the treating physician. Relapses were analyzed for up to 2 years before initiation of therapy and for the duration of the time undergoing therapy.

### Statistical Analysis

Descriptive statistics were performed on the demographic and clinical variables. Mean, median, SD, and interquartile range (IQR) were reported as appropriate. A paired 2-tailed *t* test was used to compare ARRs before and on treatment. Kaplan-Meier survival analyses were used to estimate the cumulative risk of clinical relapses on treatment, of switching treatment, and of the development of ≥2 new T2 hyperintense and/or ≥1 gadolinium-enhancing lesions on brain MRI, using the log-rank test to compare patients starting injectables (n = 89) and those starting newer DMTs (n = 14). Kaplan-Meier survival analyses were also used to estimate the cumulative risk of clinical relapses, ≥2 new MRI lesions, and EDSS score worsening for all 103 children in our cohort. In addition, we built Cox proportional hazards models to investigate the impact of DMT choice (newer vs injectables) on the risk of clinical relapses, risk of switching treatment, and risk of the development of ≥2 new T2 hyperintense and/or ≥1 gadolinium-enhancing lesions on brain MRI, after adjusting for potential confounders including sex, ethnicity, age at presentation and DMT initiation, relapse characteristics at presentation (optic neuritis, transverse myelitis, and polysymptomatic), number of relapses in the prior 6 months, and EDSS score at baseline before treatment. For each analysis performed, adjusted HRs are reported for DMT type.

For MRI outcome, we used midpoint survival analyses due to the fact that there is variation in the timing of MRI scans in clinical practice and the actual time of a new lesion developing is not known. Therefore, we used the midpoint of time between the MRI with a new lesion and previous MRI to estimate when the new lesion developed. For time to ≥2 new T2 hyperintense and/or ≥1 gadolinium-enhancing lesions analysis, we included patients if they had a baseline brain MRI within the 6 months before starting a DMT, as well as ≥1 MRI midpoint during treatment on that DMT. Results associated with a value of *p* < 0.05 were considered significant. Data were analyzed with GraphPad Prism 8 (GraphPad Software) and StataSE version 14 (StataCorp LLC, College Station, TX).

### Standard Protocol Approvals, Registrations, and Patient Consents

This study was approved by Great Ormond Street Hospital Research and Development Department (reference: 16NC10).

### Data Availability

For purposes of replicating procedures and results, any qualified investigator can request anonymized data after ethics clearance and approval by all authors.

## Results

A total of 103 children with a diagnosis of RRMS who received treatment with a DMT were identified. Median age at symptom onset was 14.0 years (IQR 12.0–14.8 years). Clinical features and patient demographics are summarized in the table. The median length of follow-up from first clinical presentation was 3.8 years (IQR 3–7 years) and from DMT initiation was 2.8 years (IQR 2.1–3.6 years).

**Table:**
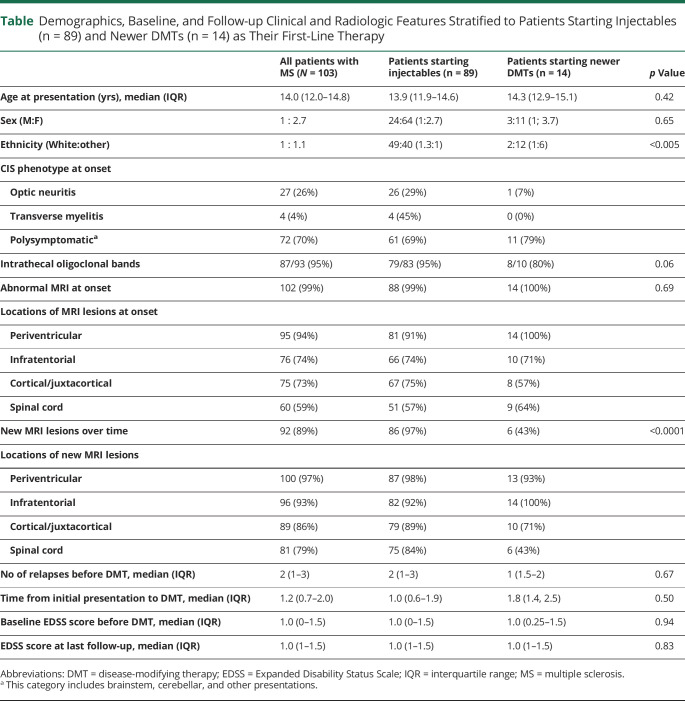
Demographics, Baseline, and Follow-up Clinical and Radiologic Features Stratified to Patients Starting Injectables (n = 89) and Newer DMTs (n = 14) as Their First-Line Therapy

**Figure 1:**
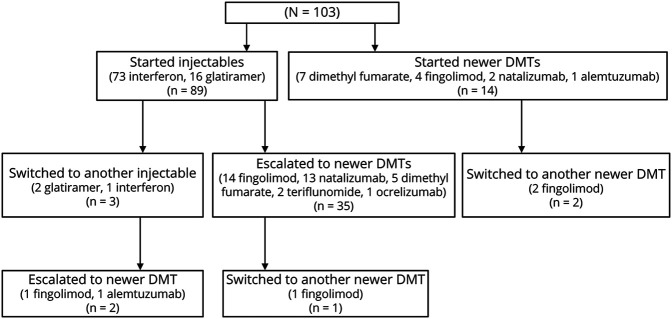
Patient Disease-Modifying Therapy (DMT) Pathway Patients were distributed across treatment centers as follows; 45 (44%) at Great Ormond Street Hospital, 25 (24%) at Evelina Children's Hospital, 18 (18%) at Birmingham Children's Hospital, 5 (5%) at Royal Manchester Children's hospital, 4 (4%) at Great North Children's Hospital, 3 (3%) at Addenbrooke's Hospital, and 3 (3%) at Alder Hey Children's Hospital. Of the 103 children included, 89 patients (86%) were started on an injectable as their first DMT (interferon-β [n = 73] and glatiramer acetate [n = 14]), and 14 (14%) were started on a newer DMT (dimethyl fumarate [n = 7], fingolimod [n = 4], natalizumab [n = 2], and alemtuzumab [n = 1]). Three of the 89 patients (3%) on injectables switched to another injectable (interferon-β [n = 1] and glatiramer acetate [n = 2]), of which 2 were then escalated to a newer DMT (fingolimod [n = 1] and alemtuzumab [n = 1]). Thirty five of 89 (39%) children who started on injectables were escalated to a newer DMT (fingolimod [n = 14], natalizumab [n = 13], dimethyl fumarate [n = 5], teriflunomide [n = 2], and ocrelizumab (n = 1)), of which 1 switched to another newer DMT (fingolimod [n = 1]). Two of the 14 patients who were started on a newer DMT as their first drug switched to another newer DMT (fingolimod [n = 2]).

Sixty-three (61%) patients were treated with 1 DMT, 37 (36%) were treated with 2 DMTs, and 3 (3%) were treated with 3 or more DMTs. Figure 1 describes the DMT pathway for all 103 patients included. Patients had a median of 2 relapses (range 1–5) before starting treatment. The median time from initial presentation to starting injectables was 1.0 years (IQR 0.6–1.9 years), whereas from initial presentation to starting a newer DMT was 1.8 years (IQR 1.4–2.5 years). Of the 305 clinical relapses reported in the cohort, 113 of these (37%) occurred while patients were on treatment.

**Figure 2:**
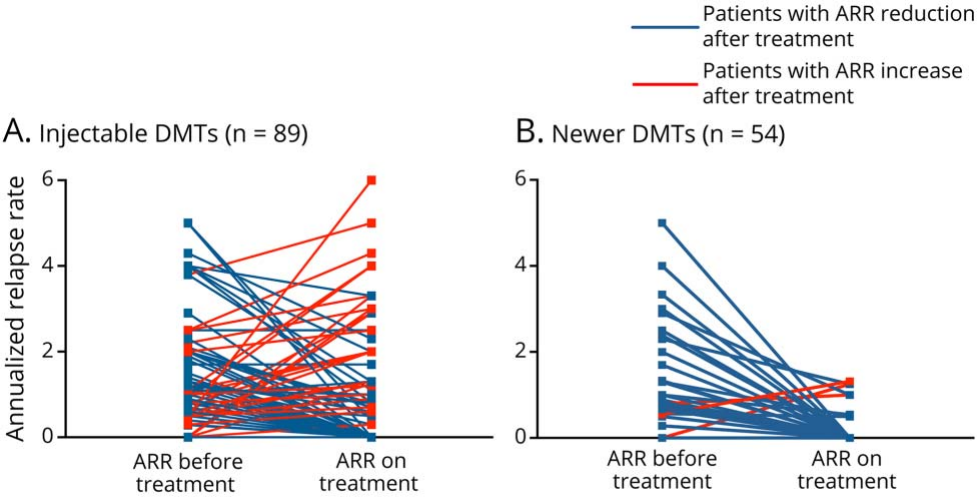
Annualized Relapse Rates Before and on Treatment Annualized relapse rates (ARRs) for patients starting injectables (A) and for patients starting or escalating to newer DMTs (B) before and on treatment: Each line corresponds to a single patient, blue lines correspond to responders (ARR reduction after treatment), and red lines correspond to nonresponders (ARR increase after treatment). Overall, the ARR was reduced from 1.9 to 1.1 while on interferon-β and glatiramer acetate (n = 92, *p* < 0.001). The ARR was reduced from 1.7 to 0.4 for newer DMTs when used as first medication (n = 14, *p* = 0.02). For newer DMTs used as 2nd- and 3rd-line treatment, the ARR was reduced from 1.6 to 0.2 (n = 40, *p* = 0.003). DMT = disease-modifying therapy.

The annualized relapse rate (ARR) reduced from 1.9 to 1.1 while on interferon-β and glatiramer acetate (n = 89, *p* < 0.001). The ARR was reduced from 1.7 to 0.4 for newer DMTs when used as first medication (n = 14, *p* = 0.02). For newer DMTs used as second- and third-line treatment, the ARR was reduced from 1.6 to 0.2 (n = 40, *p* = 0.003). Overall, when considering all patients on newer DMTs (either starting on, or escalating to newer DMTs), ARR was reduced from 1.6 to 0.3 (n = 54, *p* = 0.002). For individual newer DMTs, ARR reduction before and on treatment was as follows: 1.1 to 0.7 with dimethyl fumarate (n = 12, *p* = 0.3), 1.9 to 0.3 with fingolimod (n = 21, *p* = 0.01), 1.7 to 0.3 with natalizumab (n = 15, *p* = 0.04), 0.5 to 0.5 with teriflunomide (n = 2, *p* = 0.5), and 1.2 to 0 with alemtuzumab (n = 2, *p* = 0.1) (figure 2; figure e-1, links.lww.com/NXI/A492). For the 40 patients who escalated from injectables to newer DMTs, there was an overall reduction of ARR from 1.7 to 0.2 (*p* = 0.0001).

**Figure 3:**
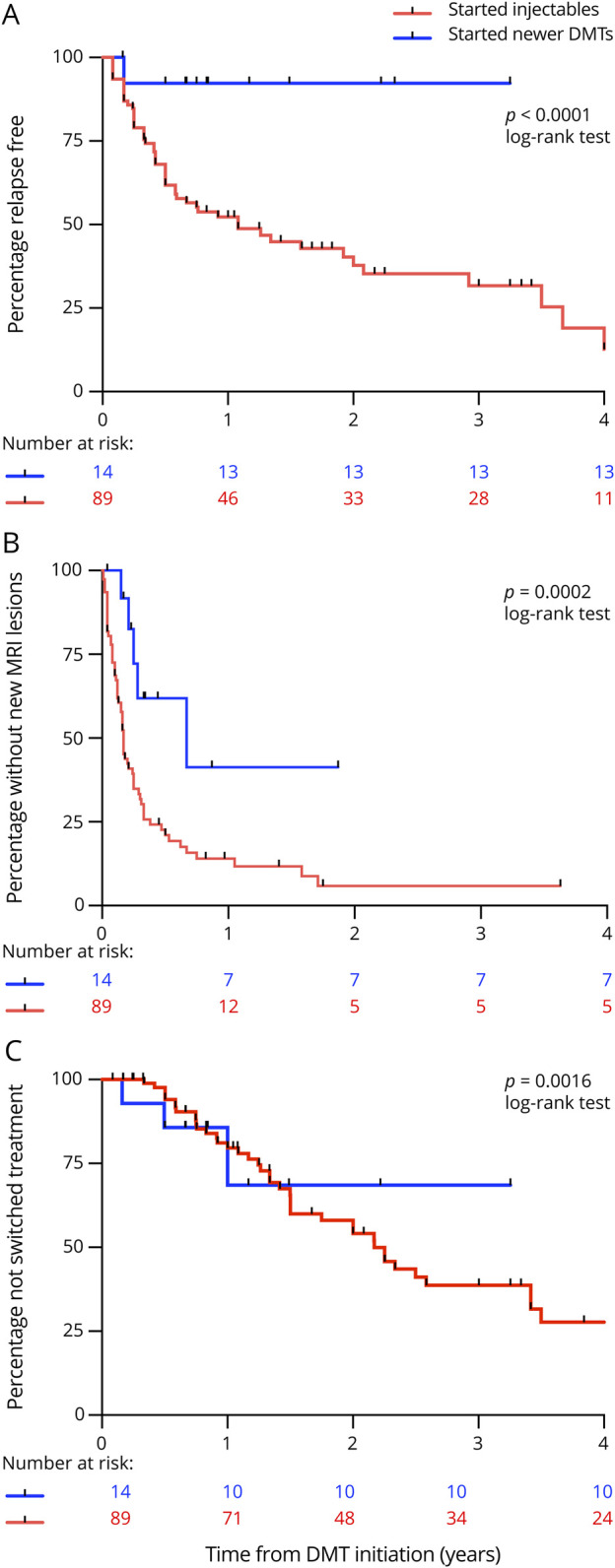
Kaplan-Meier Survival Analyses for Injectables and Newer DMTs Kaplan-Meier survival analyses estimating the cumulative risk over 24 months of clinical relapses on treatment (A), the cumulative risk of the development of ≥2 new T2 hyperintense and/or ≥1 gadolinium-enhancing lesions on brain MRI (B), and the cumulative risk of switching treatment (C) for injectables and newer disease-modifying therapies (DMTs).

Relapses on treatment were recorded in 53/89 (59.6%) of patients who had an injectables compared with 8/54 (15%) children who started on or escalated to newer DMTs. Of note, 20 patients (19.4%) who relapsed on treatment were not escalated; of these, 13 were on injectables and 7 were on newer DMTs. Kaplan-Meier survival analysis showed longer time to first relapse in children on newer DMTs. compared with injectables (log-rank *p* < 0.001) (figure 3A) and a longer time to switching treatment in children on newer DMTs compared with injectables (log-rank *p* = 0.0016) (figure 3C).

During the period, a median of 4 (range 3–10) repeated MRI scans were performed. Radiologic activity occurred in 77/89 (86.5%) of patients who had injectables compared with 26/54 (47%) who started on or escalated to newer DMTs. Kaplan-Meier survival analysis showed children on newer DMTs had longer time to developing of ≥2 new T2 hyperintense and/or ≥1 gadolinium-enhancing lesions compared with those on injectables (log-rank *p* = 0.002) (figure 3B). Median time to new radiologic activity was 2.8 years (IQR 0.5–2.0 years) on newer DMTs compared with 1.8 years (IQR 0.8–3.2 years) on injectables. Overall, clinical relapses occurred in 53/103 (51%) patients and new MRI lesions occurred in 91/103 (88%) patients at 2 years from treatment initiation. In fact, at 2 years, 38/103 (37%) patients had ≥2 new T2 hyperintense and/or ≥1 gadolinium-enhancing lesions in the absence of clinical relapses.

Baseline and follow-up EDSS scores during treatment were available for all children; the median EDSS score at baseline before treatment initiation was 1.0 (IQR 0–1.5), and at last follow-up on treatment was 1.0 (IQR 1–1.5). In total, only 10 children (9.7%) had an EDSS score ≥2 before treatment initiation, and this increased to 12 (11.7%) children at last follow-up. EDSS worsening ≥1.0 point was observed in 12 children (13%) on injectables compared with 7 children (13%) who were started or escalated to newer DMTs. Of 88 children with cognitive assessments reported, only 2 (2%) had cognitive impairment, as defined by impairment in 3 separate domains on testing.^16^

We performed multivariable analysis and adjusted our results for the variables that have been identified as potential confounders, showing that starting on injectables was associated with a twelvefold increased risk of clinical relapse (adjusted HR = 12.12, 95% CI = 1.64–89.87, *p* = 0.015) and a twofold increased risk of new radiologic activity (adjusted HR = 2.78, 95% CI = 1.08–7.13, *p* = 0.034) compared with starting on newer DMTs. For patients starting injectables, there was no increased risk of switching treatment (adjusted HR = 0.96, 95% CI = 0.28–3.29, *p* = 0.94) compared with those starting on newer DMTs.

**Figure 4:**
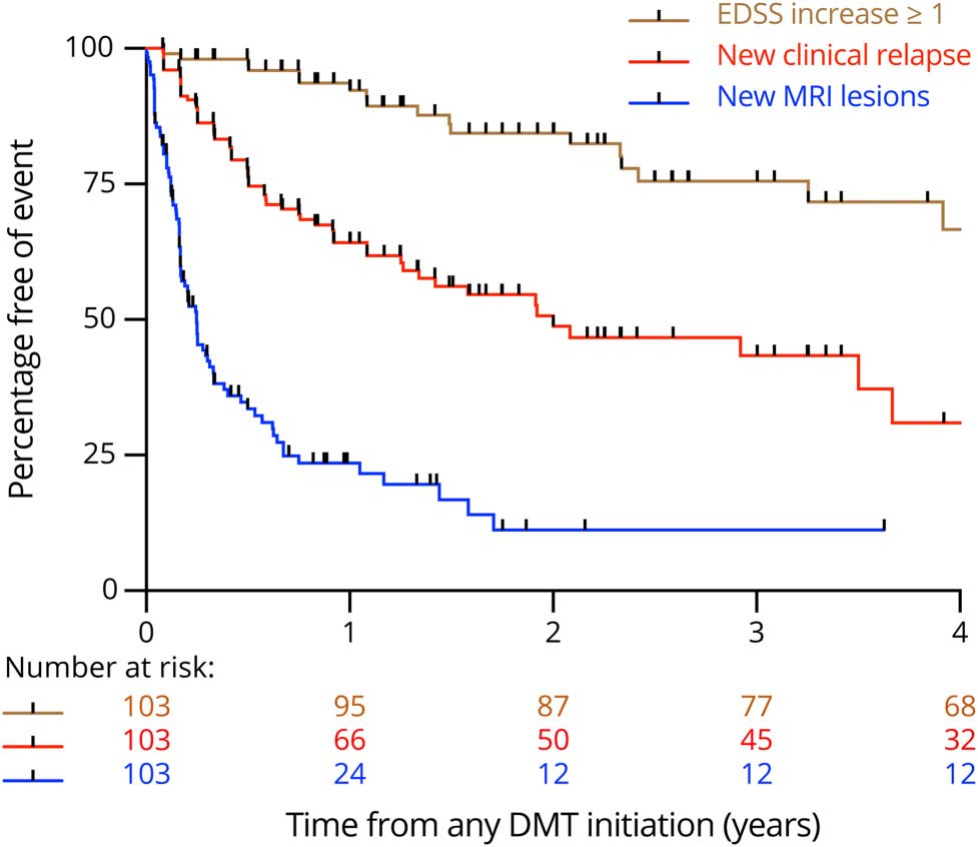
Kaplan-Meier Survival Analysis for All Patients Kaplan-Meier survival analysis estimating the cumulative risk over 24 months of clinical relapses, ≥ 2 new MRI lesions, and Expanded Disability Status Scale score worsening for all 103 children in our cohort.

Kaplan-Meier survival analysis demonstrated that the proportion of patients with ≥2 new T2 hyperintense and/or ≥1 gadolinium-enhancing lesions was higher than the proportion who had clinical relapses and EDSS score increase ≥1 throughout follow-up (figure 4).

Side effects were reported by 55/89 patients (61.8%) on injectables, of which 50 were grade 1 adverse events on the CTCAE grading scale,^15^ and 5 were grade 2. The most commonly reported side effects for injectables were flu-like symptoms (n = 19), injection site reaction (n = 16), headaches (n = 7), myalgia and fatigue (n = 5), gastrointestinal disturbance (n = 3), and derangements in liver function tests and full blood count (n = 5). Side effects were reported for 18/54 (33%) who started or were escalated to newer DMTs, of which 14 were grade 1 adverse events, and 4 were grade 2. The most commonly reported side effects for newer DMTs were derangements in liver function tests and full blood count (n = 2), gastrointestinal disturbance (n = 2), myalgia and fatigue (n = 2), and headaches (n = 1). In 5 patients, DMTs were discontinued (n = 2) or switched (n = 3) due to side effects (blood derangements n = 3; severe myalgia/flu-like symptoms n = 2).

## Discussion

In this UK-wide observational study of DMTs in children with MS, we demonstrated that treatment with newer DMTs (oral or infusions) was more effective in preventing relapses and new or gadolinium-enhancing T2 lesions compared with injectable therapies. Our results are in keeping with the first randomized control trial of fingolimod vs interferon-β in children.^12,17^ A high rate of treatment failure with injectables has been previously reported in children, ranging from 25% to 64% across studies.^18^

Over the past few decades, there has been a shift toward MRI-based measures of quantifying inflammatory activity (focal brain lesions) being the main efficacy outcomes in adult MS clinical trials. Lesion-related MRI markers provide an objective measure of the underlying MS pathology and correlate with clinical outcomes in RRMS (in particular with relapses) in the short and medium terms.^19^ Our results clearly demonstrate that children on newer DMTs took longer to new radiologic activity compared with those on injectables. However, at 2 years after treatment initiation, although only 51% patients had relapsed on treatment, 88% had developed new lesions on neuroimaging, highlighting the discrepancy between clinical activity/disease severity and radiologic features of what is largely a highly active disease phenotype in pediatrics.

With regard to measures of disability, in our cohort, children had a median EDSS score of 1.0 both at baseline before treatment initiation and at last follow-up on treatment. EDSS score ≥2 was observed in 9.7% of patients prior to treatment, which increased to 11.7% at last follow-up. Only a minority of children had evidence of disability progression (defined as EDSS worsening ≥1.0 point) on both injectables (13%) and newer DMTs (13%). A recent North American study demonstrated that children recover better from relapses than adults with MS^20^; for every 10 years of age, there was reduced EDSS recovery by 0.15 points (*p* < 0.0001). In addition, improvement in EDSS score following a relapse was seen in a larger proportion of children compared with adults (*p* = 0.006) with every 10 years of age, increasing the odds of EDSS not improving by 1.33 times. The effect of age on both heightened inflammation and neuronal plasticity^21^ influences the clinical course with better and faster recovery from relapses (even without treatments) and frequent radiologic silent activity in the absence of clinical relapse when treatments are presumably working.

This highlights that clinical outcome measures (including EDSS and ARR) are not a sensitive enough reflection of disease activity alone in children, and the current practice of waiting for the next relapse to consider treatment initiation or escalation is unsatisfactory. A cohort study of 1,555 adult patients with RRMS showed that initial treatment with fingolimod, natalizumab, or alemtuzumab was associated with a lower risk of conversion to secondary progressive MS compared with injectables.^22^ Given that brain atrophy is already evident in pediatric-onset MS at clinical presentation^5,23^ and is linked to disease activity,^24^ alternative and more sensitive pediatric specific outcome measures are therefore necessary either independent of or in addition to clinical outcome measures for accurately assessing disease activity and disease impact on the developing brain.

In our real-life clinical cohort, no pediatric-specific side effects were reported, and newer DMTs had a similar short-term safety, tolerability, and side effect profiles as in adults.^25^ Only 5 patients needed to discontinue or switch DMTs due to significant adverse events. Side effects were reported in 60% of children on injectables and 33% of children on newer DMTs. This is somewhat reassuring given the concerns of medication noncompliance in adolescence (reported as high as 47% with injectables^26,27^). Importantly, despite the reassuring safety profile, the long-term sequelae of both newer infusion and oral DMTs (including alemtuzumab, ocrelizumab, and cladribine) on the developing immune system are still unknown and will need longer-term longitudinal studies. In addition, the cumulative risk from multiple DMTs, particularly in children who are likely to require treatment for decades, needs to be considered. Fewer patients on newer DMTs switched treatment in this study, with 20 patients (19.4%) continuing on the same treatment despite disease activity. This was likely due to access and availability of DMTs at the time.

In our cohort, only 2/88 children (2%) had cognitive impairment reported in the neuropsychology testing. This is surprising given the high rates of cognitive impairment in children reported in the literature (30% of patients across studies^28,29^) and may be explained by the short follow-up period in our cohort with cognitive decline likely to occur later on in early adulthood. Data from a population-based longitudinal cohort study from the Swedish MS Registry evaluating cognitive outcome of 5,704 patients with MS revealed that 300 pediatric-onset patients had higher information processing disabilities than their counterparts with adult-onset disease, independent of age or disease duration.^30^

Limitations of this study include its retrospective nature, the small number of patients included (compared to adult MS cohorts), and the lack of long-term safety data, especially for newer DMTs. It was also not possible to blind the clinicians reviewing patients' electronic records and collecting outcome data to the DMT they received, which may have introduced measurement bias. Furthermore, as patients often switched treatment horizontally across categories (e.g., one injectable to another) or vertically (i.e., escalating from injectable to newer DMT), the different mechanisms of action of various drugs and duration to achieve effectiveness may have introduced further bias. Despite these limitations, our findings are generalizable to a broad range of patients with pediatric MS across diverse geographic areas in the United Kingdom. In addition, our findings corroborate with those from those in differing geographical locations such as the United States despite different health care systems, practice preferences, and access to certain DMTs. Furthermore, given that pediatric MS randomized controlled trials have ongoing major recruitment challenges and largely focus on establishing drug efficacy at the population level, real-world observational cohort studies such as ours can provide a valuable alternative in analyzing the multiple factors shaping treatment response and MS clinical outcome in clinical practice.

Our study adds weight to the argument for an imminent shift in practice toward the use of newer, more efficacious DMTs in the first instance. As relapses are the highest and MRI activity continues on DMTs, and this can impact on brain atrophy, which is most rapid in the first few years after onset of pediatric MS, this time period may present a critical therapeutic window for the use of highly effective therapies.

## Glossary

ARR: annualized relapse rate
DMT: disease-modifying therapy
EDSS: Expanded Disability Status Scale
HR: hazard ratio
IQR: interquartile range
MS: multiple sclerosis
RRMS: relapsing-remitting multiple sclerosis
